# Optimising Gallium-68 (⁶⁸Ga) DOTATATE PET/CT Reconstruction in Neuroendocrine Tumours: A Paired Comparison of Penalised-Likelihood (BSREM/Q.Clear) and Ordered Subset Expectation Maximisation Algorithms

**DOI:** 10.7759/cureus.95049

**Published:** 2025-10-21

**Authors:** James A Temple, Rachna Prem

**Affiliations:** 1 Urology, Epsom and St. Helier University Hospitals NHS Trust, London, GBR; 2 Nuclear Medicine, King's College London, London, GBR

**Keywords:** gallium ga 68 dotatate, hybrid iterative reconstruction, neuro endocrine tumor, nuclear medicine imaging, pet ct scan

## Abstract

Objective

The aim was to determine whether “Q.Clear” (GE Healthcare, Bayesian Penalised Likelihood (BPL) reconstruction algorithm) of Gallium-68 (⁶⁸Ga) DOTATATE PET scans at different penalisation factors (β) could improve qualitative and quantitative image parameters compared with the standard Ordered Subsets Expectation Maximisation (OSEM), VPFX reconstruction.

Methods

Twenty-five PET/CT scans performed 60 minutes after injection of 110-224 MBq (activity 153 MBq/kg) of ⁶⁸Ga-DOTATATE on a GE Discovery 710 PET/CT scanner were reconstructed using VPFX (2 iterations, 24 subsets) and Q.Clear with β values ranging from 200-1200.

A representative neuroendocrine tumour (NET) lesion and three reference regions (liver, spleen, and L3 bone marrow) were measured for standardised uptake values (SUVₘₐₓ/mean/peak/SD), signal-to-noise ratio (SNR = SUVₘₐₓ/liver SUV_SD_), and signal-to-background ratio (SBR = SUVₘₐₓ/liver SUV_mean_). A blinded qualitative assessment by a PET specialist scored image quality on a 5-point scale and evaluated the presence and severity of artefacts.

Results

BPL lesion SUVₘₐₓ and SNR were greater than VPFX for all β values (*p* < 0.05). Although BPL lesion SBR values were higher than VPFX, no β reached statistical significance. Similar patterns were observed for reference organ comparisons. Qualitative analysis showed a preference for β = 800, which yielded the best image quality with lower artefact scores.

Conclusion

BPL reconstruction of ⁶⁸Ga-DOTATATE PET data in patients with NETs improves SNR in tumour lesions and normal organs and increases SUVₘₐₓ in tumours. Combining these results with the preferred image quality at β = 800, BPL reconstruction can be considered a viable alternative for future reconstruction methods when assessing NETs.

## Introduction

Neuroendocrine tumours (NETs) comprise a biologically heterogeneous group of neoplasms characterised by neurosecretory granules, variable hormone production, and a wide spectrum of growth kinetics, ranging from indolent well-differentiated lesions to aggressive neuroendocrine carcinomas [1]. Although relatively uncommon, the reported incidence has risen, reflecting better awareness and the widespread adoption of functional imaging [1]. A unifying biological hallmark is the overexpression of somatostatin receptors (SSR), particularly subtype 2 (SSR2) and subtype 5 (SSR5), which underpins both targeted imaging and peptide receptor radionuclide therapy (PRRT) [2].

PET/CT plays a central role across the NET pathway. While fluorine-18 fluorodeoxyglucose (^18^F-FDG) is ubiquitous in oncological imaging, many well-differentiated NETs exhibit low glycolytic activity and are suboptimally characterised by FDG. In this context, gallium-68 DOTATATE (^68^Ga-DOTATATE), a somatostatin analogue with high affinity for SSR2, provides superior sensitivity for well-differentiated disease, supports accurate staging, and informs PRRT selection [2,3]. Generator-based production and favourable biodistribution have accelerated its clinical use and facilitated adoption even in centres without cyclotron facilities [3]. Typical physiological distribution includes very high splenic uptake and moderate hepatic and thyroid activity, with additional uptake in kidneys, pituitary gland, and other SSR-rich tissues, features that carry interpretive implications for lesion conspicuity and for handling background variability [2,3].

Despite the strengths of DOTATATE PET, image quality and quantitative stability are constrained by the physics of PET acquisition. Limited spatial resolution, scatter, randoms, and count-limited statistics can degrade images, particularly in the abdomen. Robust image reconstruction is therefore pivotal to deliver interpretable scans and reproducible semi-quantitative metrics. Ordered-subsets expectation maximisation (OSEM), clinically implemented by GE Healthcare as Vue Point FX (VPFX) with time-of-flight (TOF) and point-spread-function (PSF) modelling, has been the workhorse of routine PET/CT [4]. However, OSEM is vulnerable to noise amplification with iteration; clinical protocols typically stop early (pre-convergence) to control noise, at the cost of contrast recovery and small-lesion quantification [4].

Bayesian penalised-likelihood (BPL) reconstruction addresses this trade-off by adding a prior (penalty) term to the likelihood, enabling near-full convergence while constraining noise. Q.Clear (block sequential regularised expectation maximisation, BSREM) is a clinical BPL implementation in which a user-set penalisation factor (β) tunes the noise-resolution balance: lower β emphasises edges and contrast but increases noise; higher β suppresses noise at the risk of over-smoothing if excessive [5]. Properly configured, BPL approaches preserve boundaries more faithfully than post-reconstruction filtering and can yield higher contrast recovery with improved precision [5,6]. Across tracers such as FDG and fluorine-18 fluciclovine (^18^F-fluciclovine), Q.Clear has shown gains in lesion detectability, standardised uptake value (SUV) accuracy, and reader confidence compared with OSEM, but it is not yet recommended in clinical practice [6,7].

However, DOTATATE poses distinct challenges relative to FDG. High physiological uptake in the spleen and moderate liver activity mean that background texture strongly influences the detectability of small hepatic or subcapsular lesions; conversely, excessive smoothing may blunt lesion edges adjacent to high-uptake organs. Whether BPL improves DOTATATE image quality and semi-quantification in a clinically meaningful way, and which β best balances conspicuity against noise, has been less well studied than in FDG cohorts [2,6-8]. Establishing a tracer-appropriate reconstruction protocol is important for day-to-day reporting, longitudinal follow-up, and cross-centre harmonisation in audits or trials.

Rationale and knowledge gap

The routine implementation of Q.Clear in ^68^Ga-DOTATATE PET/CT has not been systematically evaluated in a paired, within-patient design across a β-ladder in NETs. Existing evidence from other tracers cannot be assumed to generalise, given DOTATATE’s receptor-mediated biodistribution and the clinical emphasis on small-lesion detection near high-uptake organs [2,6-8]. A reconstruction that materially improves signal-to-noise ratio (SNR) while maintaining stable signal-to-background ratio (SBR) and physiological appearance would be advantageous for NET imaging workflows.

Hypothesis

Compared with conventional VPFX OSEM, Q.Clear improves quantitative performance (lesion SUVₘₐₓ, SNR, and SBR stability) and qualitative image quality for ^68^Ga-DOTATATE PET/CT in NETs.

Our aims are to compare lesion SUVₘₐₓ, SNR, and SBR between VPFX and Q.Clear across multiple β values; to assess qualitative image quality and artefact burden for each reconstruction; to identify a β that optimally balances lesion conspicuity and background smoothness for routine DOTATATE imaging; and to evaluate inter-observer reproducibility of quantitative measurements under each reconstruction.

By addressing these aims in a paired design using clinical datasets, this study seeks to define a pragmatic, tracer-specific reconstruction setting for DOTATATE PET/CT that enhances lesion visibility and stabilises semi-quantification, thereby supporting confident reporting, consistent follow-up, and potential harmonisation across centres.

## Materials and methods

Study design and setting

We performed a case-controlled, paired reconstruction comparison study using clinical ^68^Ga-DOTATATE PET/CT examinations for NETs. Twenty-five consecutive scans acquired for routine care were included. No additional imaging was undertaken for research; analyses used exported clinical datasets. Demographic or tumour subtype stratification was not applied for reconstruction comparisons, which were conducted within-patient across all algorithms. Ethical approval was obtained from the relevant institutional review teams.

Radiopharmaceutical and uptake protocol

Patients received an intravenous bolus of ^68^Ga-DOTATATE, with administered activities between 109.9 and 224.4 MBq (approximately 153 MBq/kg), adjusted to patient habitus. Imaging commenced after an approximately 60-minute uptake period to allow receptor binding and tracer distribution to stabilise.

PET/CT acquisition

Whole-body PET/CT imaging (skull base to mid-thigh) was performed in 3D mode on a GE Discovery 710 PET/CT scanner. The CT component provided anatomical localisation and attenuation correction. Vendor-standard corrections (normalisation, attenuation, scatter, and randoms) were applied. Acquisition coverage and bed positions were kept identical across reconstructions to allow paired, slice-matched analyses.

Reconstruction protocols

Each raw dataset was reconstructed twice using distinct pipelines. The first used conventional OSEM (VPFX) with two iterations and 24 subsets, incorporating TOF and point-spread function (PSF) modelling, as per clinical defaults. The second employed BPL (Q.Clear, block sequential regularised expectation maximisation (BSREM)), generating series at β = 200, 400, 600, 800, 1000, and 1200. No post-reconstruction filtering beyond algorithmic regularisation was applied.

The same PET sinograms and CT attenuation maps were used for all reconstructions, and for each patient, the full β-ladder was generated in the same session to prevent pre-processing variability.

Image review platform and display standardisation

All series were reviewed on the Hermes Hybrid Viewer (Fusion mode). Colour scales and windowing followed institutional presets and were held constant for each patient across all reconstructions. Axial slice indices for quantitative sampling were recorded on the VPFX series and replicated on each Q.Clear series.

Quantitative analysis

Two observers (trained medical students) performed measurements after a joint calibration on five training cases under the supervision of a nuclear medicine physician. The remaining 20 cases were analysed independently.

Lesion selection and volume of interest (VOI) placement

**Figure 1:**
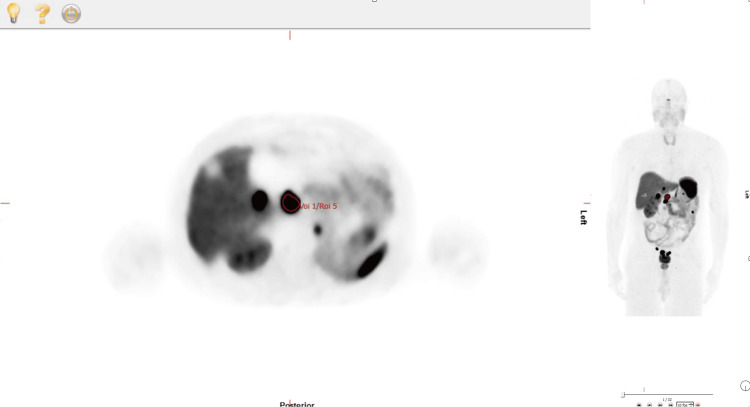
Example of a segmented lesion on a patient scan. This image demonstrates the result when the VOI tool is correctly configured. The left image shows a transverse plane slice, and the right image presents a whole-body view. VOI: Volume of interest.

For each patient, one representative NET lesion was selected using clinical correlation and visual uptake characteristics. A semi-automatic VOI was applied using a 40% of lesion SUVₘₐₓ threshold; the hottest focus nearest the click position was segmented. Axial indices and lesion coordinates were recorded to ensure identical placement across reconstructions (Figure 1).

Reference organs and VOI definitions

**Figure 2:**
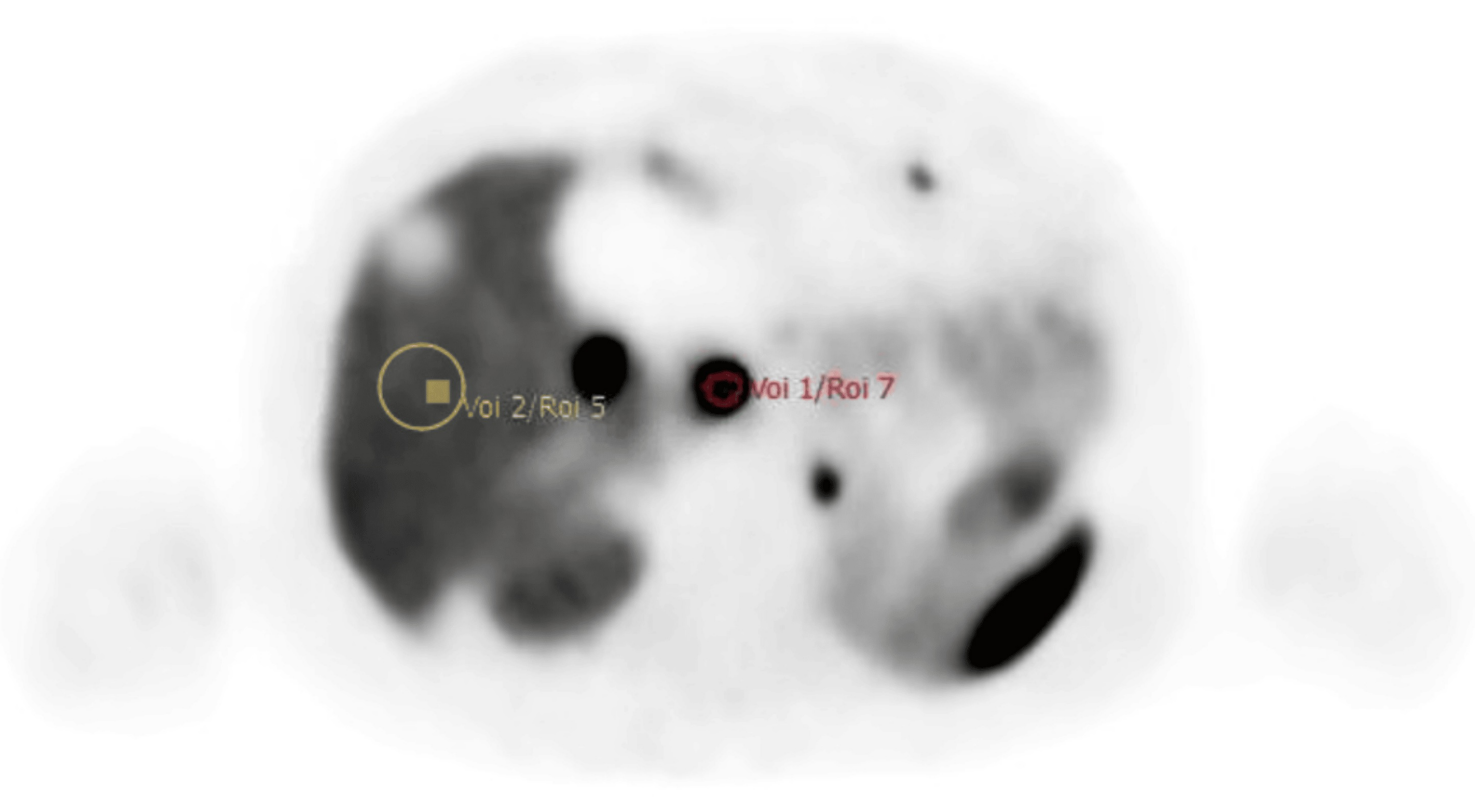
Segmentation of the lesion using the VOI tool, in combination with the VS tool for liver delineation. VOI: Volume of interest; VS: Volume segmentation.

Three reference regions were sampled to reflect a range of physiological uptake: Liver: 3 cm³ spherical VOI in uninvolved parenchyma, avoiding vessels and metastases. Spleen: 3 cm³ spherical VOI centred within splenic parenchyma, avoiding the hilum. Bone marrow: 2 cm³ spherical VOI within the L3 vertebral body, avoiding cortical bone (Figure 2).

Where minor adjustments were required to remain within the same tissue compartment across different series, spheres were minimally translated while preserving the recorded slice index.

Metrics and derived indices

For each lesion and organ VOI, we exported SUVₘₐₓ, standardised uptake value mean (SUV_mean_), standardised uptake value peak (SUV_peak_), and standardised uptake value standard deviation (SUV_sd_). From these, two indices were derived as follows:

\[\mathrm{SNR}=\frac{\mathrm{SUV}_{\max}^{\text{lesion}}}{\sigma\!\left(\mathrm{SUV}^{\text{liver}}\right)}\]

\[\mathrm{SBR} \;=\; \frac{\mathrm{SUV}_{\max}^{\text{lesion}}}{\mathrm{SUV}_{\text{mean}}^{\text{liver}}}\]

For organ-specific explorations, the numerator was replaced with the corresponding organ SUVₘₐₓ. All values were entered into a pre-formatted spreadsheet with one row per patient and seven columns (VPFX plus six β levels), with embedded formulae for summary statistics and paired tests.

Measurement quality control

VOIs were centred well within target structures to mitigate partial-volume and misregistration effects. Liver spheres were repositioned if unsuspected focal uptake or metastasis was identified. No additional smoothing or denoising was applied beyond each reconstruction’s intrinsic regularisation.

Qualitative image assessment

A nuclear medicine physician with more than 20 years of PET/CT experience performed a blinded qualitative review. For each reconstruction series: Overall image quality (five-point Likert scale: 1 = poor, 5 = excellent), artefact presence (binary: 0 = absent, 1 = present), and artefact severity (three-point ordinal scale: 1 = mild, 3 = severe). Per-series mean scores were calculated across the cohort and used to rank qualitative preference.

Inter-observer reproducibility

After the joint calibration set, the two observers independently analysed the remaining cases. Intraclass correlation coefficients (ICC) were computed for lesion and organ SUVₘₐₓ, SNR, and SBR to assess reproducibility. ICC values greater than 0.90 were prespecified as indicating excellent agreement.

Statistical analysis

Analyses were conducted in Microsoft Excel using paired, two-sided t-tests (α = 0.05), comparing each Q.Clear β level with VPFX (within-patient). We report the t statistic and degrees of freedom (df = 24) alongside corresponding p-values for these comparisons. No chi-square tests or ANOVA were used for the primary paired analyses; therefore, χ² and F values are not applicable. ICCs were computed for inter-observer reproducibility. Box-and-whisker plots were generated from the spreadsheet tables to illustrate value distributions across reconstructions.

## Results

Quantitative results

Lesion Metrics

Across all reconstructions using the BPL algorithm (Q.Clear), lesion SUVₘₐₓ and SNR were higher than those obtained with the conventional OSEM implementation (VPFX). For SUVₘₐₓ, every penalisation factor (β200-β1200) exceeded VPFX with statistical significance (p < 0.05). The highest observed lesion SUVₘₐₓ occurred at β200 (152.54), while the overall mean lesion SUVₘₐₓ across patients and reconstructions was 20.42. Lesion SNR was also higher for all Q.Clear reconstructions. At β200, the increase reached marginal significance with t(24) = 2.07, p = 0.049; higher β levels remained significant. Lesion SBR showed a small rise at β200 that did not reach significance (t(24) = 1.57, p = 0.13). For all subsequent β levels, comparisons were not significant with p > 0.19, corresponding to |t(24)| < 1.35; distributions remained tightly clustered around VPFX.

To present these comparisons clearly, Table 1 summarises the directionality and significance for each lesion metric at each β value. The table reports whether the metric increased or remained essentially unchanged relative to VPFX, alongside the statistical outcome for the paired comparison.

**Table 1:** Lesion comparisons between Q.Clear and VPFX using paired t-tests (df = 24). SUVₘₐₓ: Standardised Uptake Value Maximum; SNR: Signal-to-Noise Ratio; SBR: Signal-to-Background Ratio; OSEM: Ordered-Subsets Expectation Maximisation; VPFX: Vendor implementation of OSEM by GE Healthcare; Q.Clear: Bayesian Penalised Likelihood (BPL) Reconstruction Algorithm; df: Degrees of Freedom.

β	Metric	Direction vs VPFX	t (df = 24)	p-value	Significant
200	SUVₘₐₓ	Higher	> 2.06	< 0.05	Yes
400	SUVₘₐₓ	Higher	> 2.06	< 0.05	Yes
600	SUVₘₐₓ	Higher	> 2.06	< 0.05	Yes
800	SUVₘₐₓ	Higher	> 2.06	< 0.05	Yes
1000	SUVₘₐₓ	Higher	> 2.06	< 0.05	Yes
1200	SUVₘₐₓ	Higher	> 2.06	< 0.05	Yes
200	SNR	Higher	2.07	0.049	Yes
400	SNR	Higher	> 2.06	< 0.05	Yes
600	SNR	Higher	> 2.06	< 0.05	Yes
800	SNR	Higher	> 2.06	< 0.05	Yes
1000	SNR	Higher	> 2.06	< 0.05	Yes
1200	SNR	Higher	> 2.06	< 0.05	Yes
200	SBR	Higher	1.57	0.13	No
400	SBR	~ No change	< 1.35	> 0.19	No
600	SBR	~ No change	< 1.35	> 0.19	No
800	SBR	~ No change	< 1.35	> 0.19	No
1000	SBR	~ No change	< 1.35	> 0.19	No
1200	SBR	~ No change	< 1.35	> 0.19	No

**Figure 3:**
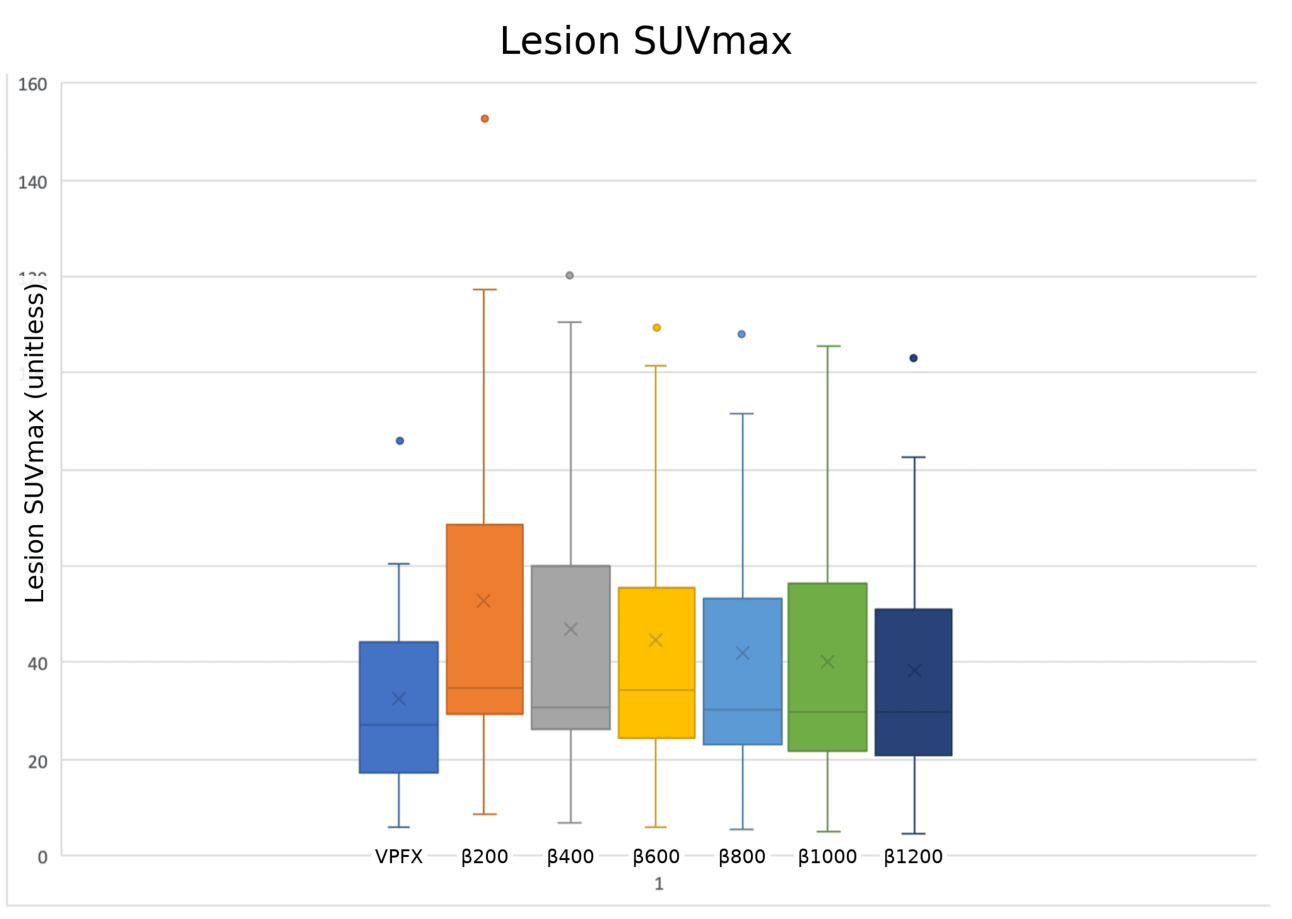
Box-and-whisker plot showing lesion standardised uptake value maximum. From left to right: VPFX, β200, β400, β600, β800, β1000, and β1200. Dots represent outliers in each distribution. SUVₘₐₓ: Standardised Uptake Value Maximum.

**Figure 4:**
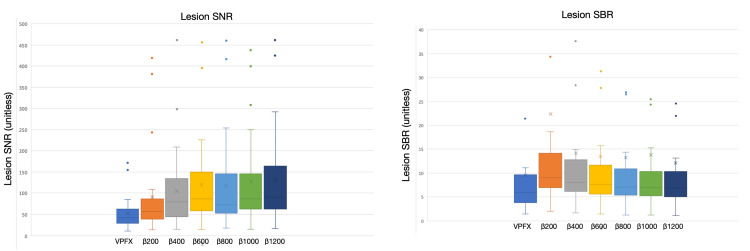
Left: Box-and-whisker plot showing lesion signal-to-background ratio (SBR). Right: Box-and-whisker plot showing lesion signal-to-noise ratio (SNR). From left to right: VPFX, β200, β400, β600, β800, β1000, and β1200. Dots represent outliers in each distribution.

Descriptive distributions (box-and-whisker plots) illustrated these trends: lesion SUVₘₐₓ values for all Q.Clear reconstructions lay above those for VPFX, with the greatest separation at lower β values and some convergence at higher β as smoothing increased. SNR distributions exhibited an overall upward shift with increasing β, reflecting progressive noise suppression; a small deviation at β800 was noted in the lesion SNR trend, yet values remained significantly higher than VPFX. In contrast, SBR distributions were tightly clustered around the VPFX baseline across all β values. The slight elevation at β200 did not reach the significance threshold (p = 0.13), and all subsequent comparisons were non-significant (p > 0.19), indicating that lesion-to-liver contrast remained essentially stable across reconstructions (Figures 3-4).

Reference Organ Metrics

**Figure 5:**
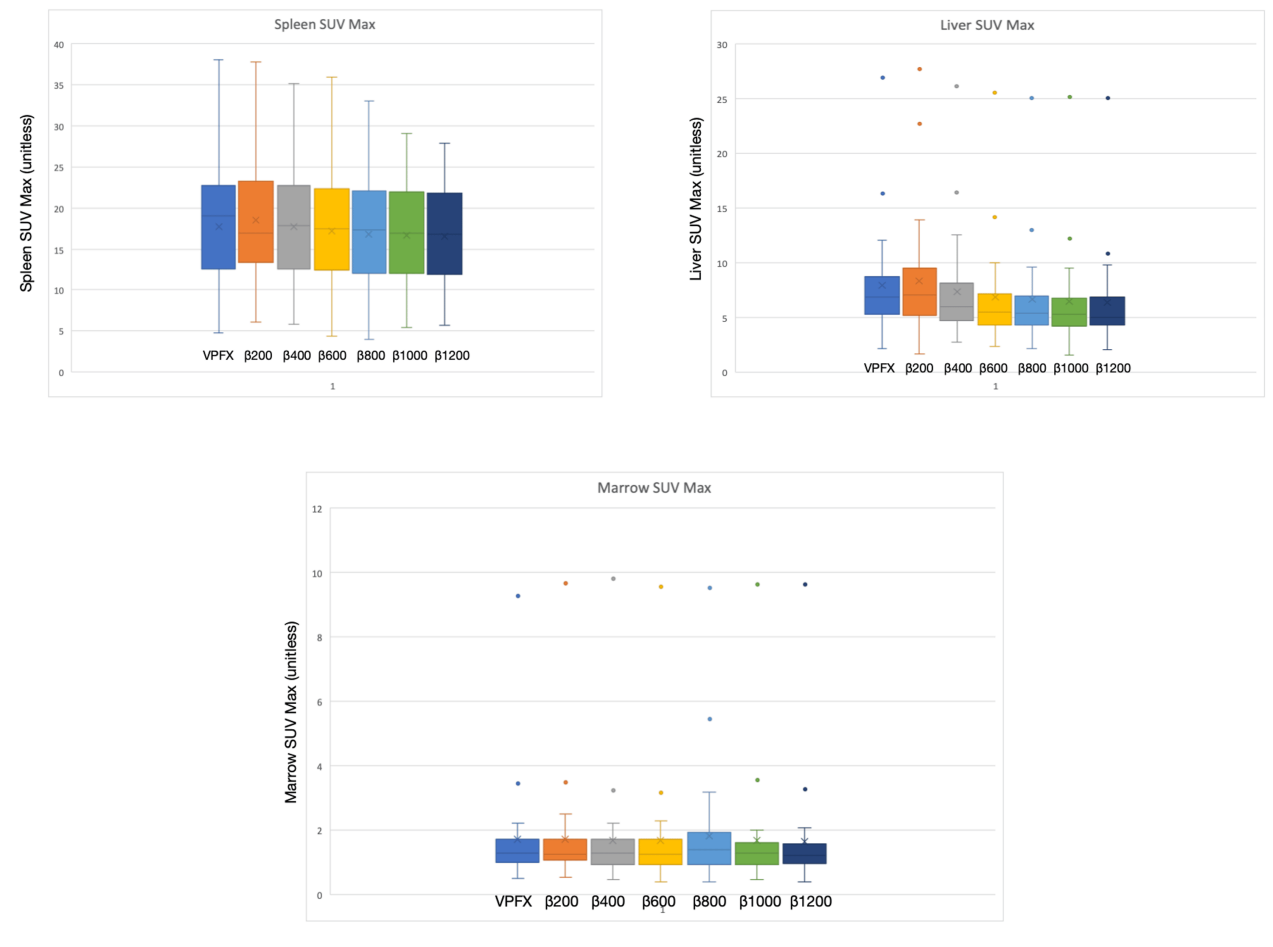
Standardised uptake value maximum of the three reference organs: spleen, liver, and bone marrow. From left to right: VPFX, β200, β400, β600, β800, β1000, and β1200.

Reference organ SUVₘₐₓ (liver, spleen, and bone marrow) did not show significant differences between VPFX and Q.Clear reconstructions across the β range. This stability in raw organ uptake contrasted with consistent improvements in SNR at moderate-to-high β levels, indicating that penalisation primarily reduced background variability (the denominator of SNR) rather than altering mean uptake (Figure 5).

**Figure 6:**
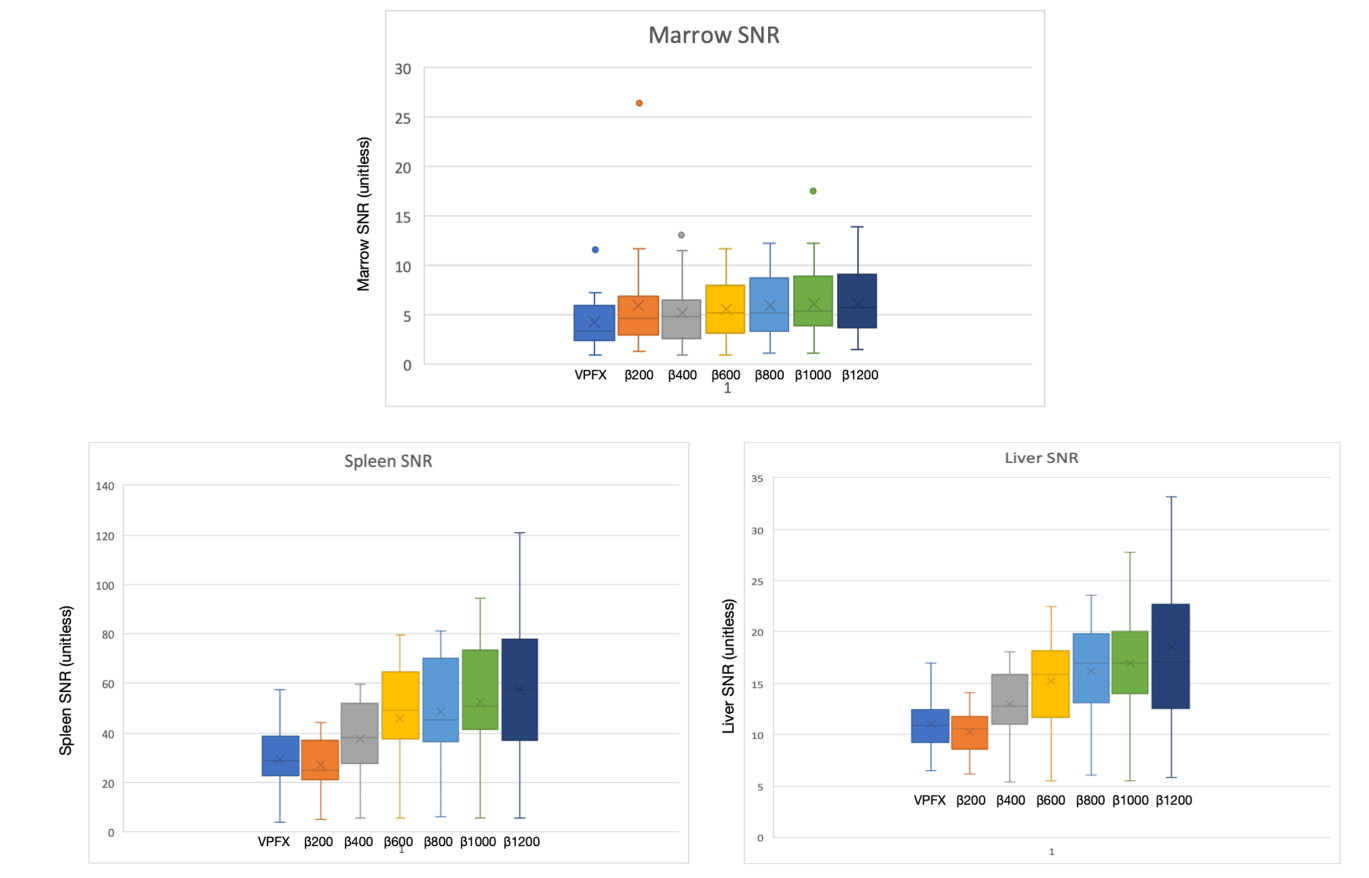
Signal-to-noise ratio (SNR) of the three reference organs: bone marrow, spleen, and liver. From left to right: VPFX, β200, β400, β600, β800, β1000, and β1200.

For the liver, SNR increased incrementally from β400 through β1200, with paired comparisons versus VPFX reaching statistical significance across this range (p values between 1.63 × 10^-^⁶ and 0.0086). For the spleen, SNR at β200 was lower than VPFX but increased thereafter and became significantly higher than VPFX from β400 upward (p = 4.48 × 10⁻⁵ to 0.037). For bone marrow, SNR demonstrated a threshold effect, becoming significantly higher than VPFX from β600 through β1200 (p = 0.017 to 0.036). These organ-wise SNR patterns were reflected in the corresponding box-and-whisker plots, which showed upward shifts in central tendency and compression of dispersion at the β levels where significance was reached (Figure 6).

**Figure 7:**
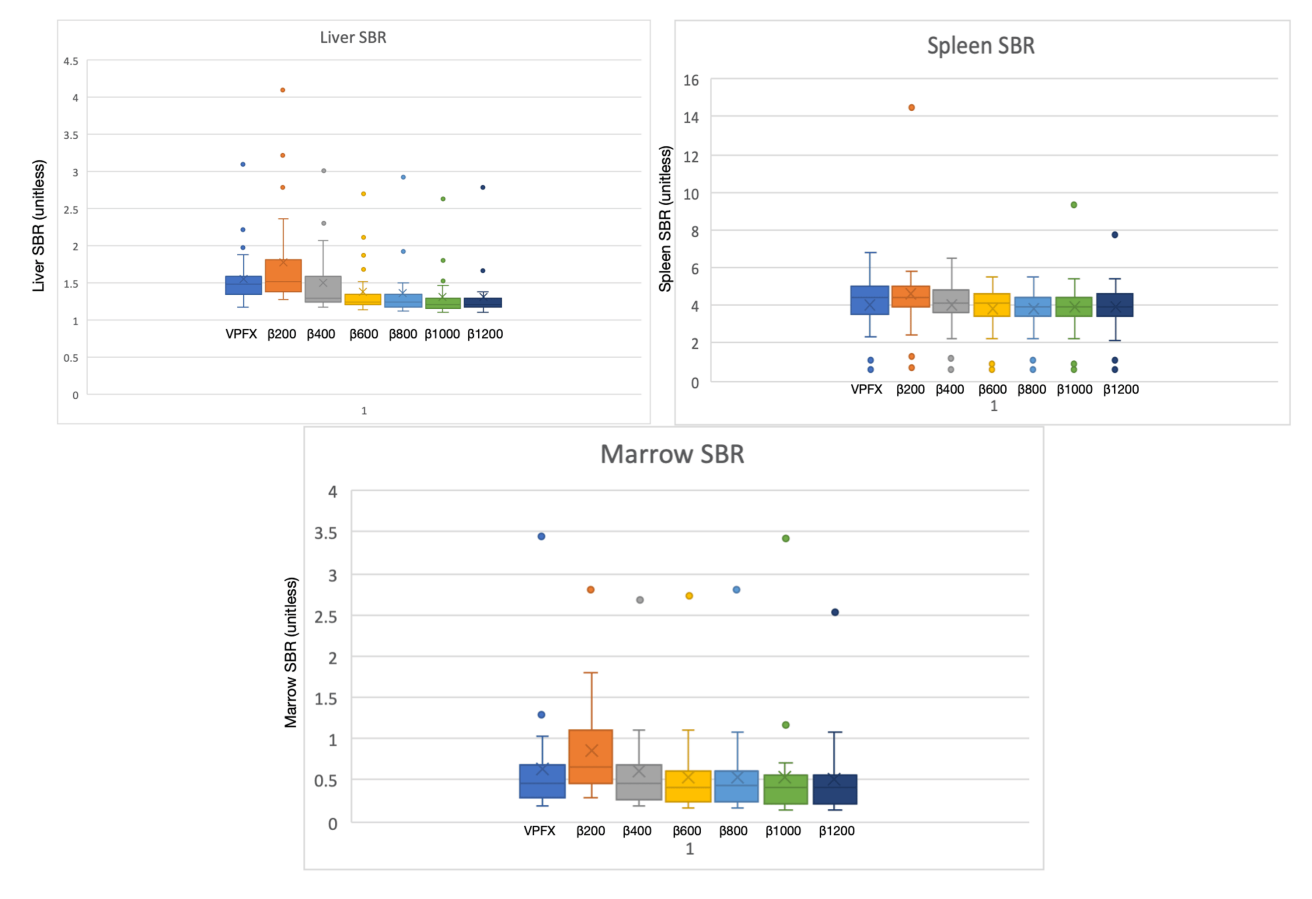
Signal-to-background ratio (SBR) of the three reference organs: bone marrow, spleen, and liver. From left to right: VPFX, β200, β400, β600, β800, β1000, and β1200.

SBR for reference organs did not favour either reconstruction method; β200 yielded the highest SBR for each organ, but the differences were not statistically significant. SBR values converged at higher β levels, mirroring lesion behaviour (Figure 7).

Table 2 summarises SNR outcomes by organ at each β level, and Table 3 summarises SBR behaviour.

**Table 2:** Reference-organ signal-to-noise ratio (SNR) compared with VPFX: significant β ranges with corresponding t and p values.

Organ	Significant β Range	t (df = 24) Range	p-value range	Significant
Liver	β400-β1200	2.86-6.30	0.0086-1.63 × 10⁻⁶	Yes
Spleen	β400-β1200	2.21-4.97	0.037-4.48 × 10⁻⁵	Yes
Bone marrow	β600-β1200	2.22-2.57	0.036-0.017	Yes

**Table 3:** Reference-organ signal-to-background ratio (SBR) compared with VPFX: summary of paired t-tests.

Organ	Across β200-β1200	Significant
Liver	~ No change	No
Spleen	~ No change	No
Bone marrow	~ No change	No

Taken together, the reference organ analyses indicate that Q.Clear enhances SNR in normal tissues at moderate-to-high β values without materially altering SBR. This pattern is consistent with stronger noise control while maintaining comparable organ-to-background contrast, a desirable feature for whole-body interpretation and lesion detection adjacent to high-uptake organs. The stability of organ SUVₘₐₓ across reconstructions also supports the view that penalised likelihood reconstruction does not bias mean organ uptake, instead chiefly reducing variance within the parenchyma.

Qualitative Image Assessment

The qualitative evaluation by an experienced nuclear medicine physician corroborated the quantitative improvements. Mean overall image quality scores were higher for Q.Clear than for VPFX at β400 and above, peaking at β600 and β800 (mean 3.9 on a five-point scale). Artefact burden was highest at β200 and progressively diminished with increasing β, reaching zero mean artefact presence and zero mean severity at β800-β1200. VPFX had a low but non-zero artefact rate and lower mean image quality than β600-β1200. The maximum quality score awarded (5/5) was achieved by all Q.Clear reconstructions from β400 upward; VPFX and β200 achieved a maximum of 4/5. These results indicate that β800 provided the best balance between image sharpness and noise suppression, combining top-tier perceived quality with minimal artefacts (Table 4).

**Table 4:** Qualitative image assessment by reconstruction.

Reconstruction	Mean Overall Quality (1-5)	Maximum Score Given (1-5)	Mean Artefact Presence (0-1)	Mean Artefact Severity (1-3)
VPFX	2.7	4	0.3	0.3
β200	2	4	1	1.8
β400	3	5	0.6	0.7
β600	3.9	5	0.1	0.1
β800	3.9	5	0	0
β1000	3.6	5	0	0
β1200	3.4	5	0	0

Qualitative preferences aligned with the quantitative patterns: lesion SUVₘₐₓ and SNR gains translated into higher perceived quality, while the progressive reduction in background noise at higher β values eliminated artefacts. The slight deviation in lesion SNR around β800 did not compromise subjective grading; indeed, β800 achieved the joint-highest quality with the lowest artefact scores, supporting it as a practical default for clinical reconstruction in ^68^Ga-DOTATATE PET/CT.

Inter-Observer Reproducibility

Inter-observer agreement for quantitative measurements was excellent across lesions and all reference organs. ICC were ≥0.91 for all parameters, with the highest agreement seen for SUVₘₐₓ (reflecting direct extraction from VOI data without derived ratios) and slightly lower, albeit still excellent, agreement for SNR and SBR ratio, which incorporate multiple measured values. The marrow SUVₘₐₓ had the highest ICC (0.98), likely reflecting the constrained VOI placement within the vertebral body. The lowest ICC was observed for lesion SBR (0.91), consistent with compounded variability when calculating ratios that include background metrics. The complete ICC summary is presented in Table 5.

**Table 5:** Inter-observer intraclass correlation coefficients (ICC). ICC: Intraclass Correlation Coefficient; SUV_max_: Standardised Uptake Value Maximum; SNR: Signal-to-Noise Ratio; SBR: Signal-to-Background Ratio.

Structure	SUV_max_ ICC	SNR ICC	SBR ICC
Lesion	0.96	0.92	0.91
Liver	0.97	0.94	0.95
Spleen	0.97	0.95	0.94
Bone marrow (L3)	0.98	0.95	0.95

Overall, the reproducibility analysis supports the robustness of the measurement protocol across observers, particularly for SUVₘₐₓ. The strong agreement for SNR and SBR further indicates that the use of liver-based background metrics (SUV_sd_ and SUV_mean_) is stable when VOI placement is standardised. These reproducibility data, combined with the quantitative and qualitative findings above, reinforce the conclusion that Q.Clear improves lesion conspicuity and perceived image quality without sacrificing measurement stability.

Summary of Key Findings

Lesion SUVₘₐₓ and SNR were higher for all Q.Clear reconstructions than for VPFX, with statistical significance for each β level; SBR did not differ significantly. Reference organ SNR increased at moderate-to-high β values (liver from β400; spleen from β400; marrow from β600) with significant p-values, while SBR remained unchanged. Qualitative assessment favoured Q.Clear, with β800 providing the best overall balance (highest mean quality and negligible artefact burden). Inter-observer agreement was excellent for all parameters (ICC ≥0.91), highest for marrow SUVₘₐₓ (ICC 0.98) and lowest for lesion SBR (ICC 0.91).

These results, derived from paired reconstructions of the same clinical datasets and summarised in the tables above, demonstrate consistent advantages of penalised likelihood reconstruction for ^68^Ga-DOTATATE PET/CT in NET imaging. In this cohort, penalisation around β800 yielded the most favourable balance of lesion conspicuity, background suppression, and subjective image quality, while preserving measurement reproducibility across observers.

## Discussion

Principal findings and interpretation

In this paired, within-patient evaluation of reconstruction strategies for ^68^Ga-DOTATATE PET/CT in NETs, penalised-likelihood (BSREM; Q.Clear) consistently outperformed OSEM (VPFX) for the two metrics that matter most to readers at the console, lesion standardised uptake value maximum (SUVₘₐₓ) and lesion SNR, while lesion SBR remained essentially unchanged. Reference-organ SUVₘₐₓ values (liver, spleen, marrow) were stable across algorithms, but their SNR increased significantly from moderate β values onward, yielding smoother parenchyma without distorting physiological means. Blinded qualitative review aligned with these quantitative patterns, favouring β600-β800 and identifying β ≈ 800 as a practical balance point between contrast and noise. Inter-observer agreement was excellent across lesion and organ metrics, supporting the reproducibility of the workflow and mitigating concerns that penalisation might introduce operator dependence.

These findings are coherent with reconstruction theory: OSEM’s subset acceleration and early iteration stopping limit contrast recovery, particularly for small or low-count structures, while BSREM’s prior term permits near-full convergence and shrinks variance preferentially in relatively uniform regions, thereby preserving edges [9-11]. For DOTATATE specifically, where physiology imposes very high splenic and moderate hepatic uptake, improved SNR without a systematic shift in lesion-to-liver ratios is clinically attractive. Readers gain cleaner backgrounds and crisper borders for small foci near high-uptake organs, yet familiar interpretive anchors (e.g., “tumour uptake greater than liver”) remain valid [12-14].

Why SNR rose while SBR held steady

Apparent tension between rising SNR and stable SBR dissolves once the definitions are considered. SNR divides a lesion signal by a variance term from the background (here, liver SUVsd). Penalisation is designed to shrink variance; thus, SNR improves even if mean background and lesion values remain steady. SBR, by contrast, is a ratio of means (lesion ÷ liver). Because both numerator and denominator reflect receptor-mediated DOTATATE uptake, modest algorithm-dependent changes tend to move in tandem, conserving the ratio. In short, BSREM makes lesions easier to see (SNR) without changing their relative brightness to the liver (SBR), which is exactly what a cautious imaging service seeks when standardising protocols [12-15].

Choosing β for DOTATATE: tracer-specific optimisation

Our results converge on β ≈ 800 as a pragmatic default for routine ^68^Ga-DOTATATE PET/CT. This β is higher than ranges often cited for ^18^F-FDG or ^18^F-fluciclovine in other disease settings and reflects tracer physics and biodistribution. DOTATATE’s strong, structured physiological uptake (spleen >> liver > marrow) places a premium on background stabilisation without edge loss [16-18].

Lower β values (e.g., β200) deliver the highest SUVs but at the cost of mottled backgrounds and reader-perceived artefacts, whereas very high β values smooth aggressively, flattening subtle borders and reducing SUV peaks. Around β800, the SNR plateau and qualitative “sweet spot” coincide, suggesting diminishing returns for further penalisation. There are other tracers, such as DOTATOC and DOTANOC, that are similar to DOTATATE; however, as they were not used, no comment can be made on whether these settings can be applied to them.

From an operational standpoint, a fixed β simplifies practice: technologists need not tune reconstruction case-by-case; radiologists become familiar with a predictable noise texture; and physicists can maintain harmonised quality assurance (QA). For multicentre work, a standard β combined with stable acquisition parameters (uptake time, bed time, and TOF settings) enables tighter equivalence in pooled analyses [19,20].

Clinical implications

Staging, Restaging and PRRT Selection

DOTATATE PET/CT underpins staging and restaging for well-differentiated NETs, guides eligibility for PRRT, and monitors treatment response. The combination of higher lesion SUVₘₐₓ and cleaner liver/splenic backgrounds at β ≈ 800 supports detection of small hepatic subcapsular deposits, mesenteric nodal disease, and trabecular bone foci that are prone to be masked by variance on under-regularised reconstructions. Because SBR is stable, qualitative thresholds used in practice (e.g., tumour ≥ liver) should not be up- or down-shifted by reconstruction choice, reducing the risk of reconstruction-driven misclassification at the margins of PRRT eligibility [12,21].

Follow-up Comparability and Language in Reports

Longitudinal consistency hinges on reducing algorithm-driven volatility. With a fixed β, modest (10-20%) SUV changes are less likely to be reconstruction artefacts and more likely to reflect true biological variation. Reports can explicitly state the reconstruction method and β value (e.g., “BSREM, β800”) in the technical section; this small addition helps downstream clinicians and external reviewers interpret subtle serial changes with confidence. In multidisciplinary meetings, cleaner backgrounds also reduce equivocal language, streamlining decisions when margins are tight (e.g., “observe vs treat”).

Reading Efficiency and Education

Qualitative gains around β600-β800 translate into fewer false-positive speckles to adjudicate and less time spent toggling window levels to determine whether a hotspot represents noise. For trainees, a steady background texture accelerates pattern recognition; for MDT discussions, uniform parenchyma makes tiny foci easier to demonstrate and reach consensus on.

Relationship to Prior Literature

Our lesion-level gains in SUVₘₐₓ and SNR align with prior experience from ^18^F-FDG and ^18^F-fluciclovine cohorts, where BSREM improved contrast recovery and reader confidence compared with OSEM [16,22]. The stable SBR we observed fits DOTATATE’s biology, lesion and liver both concentrate tracer via somatostatin receptor subtype 2 (SSR2), whereas FDG studies sometimes show SBR gains because smoothing disproportionately reduces background glycolytic activity [16,22,23]. The optimal β is tracer- and task-specific: values suitable for FDG nodal staging or fluciclovine imaging in prostate bed recurrence need not be directly applied to DOTATATE. Few prior reports have systematically evaluated a β-ladder in DOTATATE with paired reconstructions; this study helps fill that gap and provides a practical default to trial in routine clinical service [17,18,24].

Methodological reflections

Within-Patient Pairing and VOI Discipline

The paired design avoids common confounders (habitual uptake, timing, body habitus). Locking volumes of interest (VOIs) to identical slices across reconstructions minimised partial-volume drift. The excellent intraclass correlation coefficients (ICCs) across lesion and organ metrics indicate that, once VOI practice is standardised, the advantages of BSREM are observer-robust rather than the product of a single analyst’s style.

Qualitative Blinding and Recognition Bias

Although the reader was blinded to series labels, experienced observers can often infer reconstruction from texture. That said, the co-movement of objective metrics (SUVₘₐₓ/SNR) and subjective scores, along with the monotonic drop in artefact rates with increasing β, reduces the likelihood that recognition alone explains the observed pattern. Future work could include multiple readers, randomised series order, and forced-choice preference testing to quantify agreement and confidence intervals for perceived image quality [25].

Corrections, Calibration, and the Rest of the Chain

Reconstruction is one link in a chain that includes attenuation, scatter, and randoms correction, as well as system calibration. X-ray-based attenuation correction stabilises SUVs compared with transmission sources, especially in larger patients. Model-based scatter and robust randoms correction mitigate variance sources, while calibration and dead-time characterisation uphold longitudinal SUV integrity [26-28]. A fixed β complements, but does not replace, these fundamentals. Centres adopting BSREM should ensure their quality assurance (QA) schedule remains intact after software updates and confirm that β still “lands” on the same quality/SNR balance following upgrades.

Organ-Specific Behaviour

SNR significance thresholds differed by organ (earlier in liver and spleen, later in marrow), plausibly due to baseline counts and VOI sizes. This heterogeneity argues for a single standard β that performs adequately across organs, rather than impractical organ-specific tuning. The net effect, smoother but physiologically faithful organs, helps adjudicate tiny lesions abutting high-uptake parenchyma.

Strengths and limitations

Strengths include the paired design, a full β-ladder generated from identical raw data, disciplined VOI placement, and excellent reproducibility. The agreement between quantitative and qualitative outcomes supports face validity for β ≈ 800 as a routine default.

Limitations merit acknowledgement. First, the sample size (n = 25), while typical for reconstruction studies, limits precision and precludes subgroup analyses by primary site, grade, or metastatic pattern. Second, lesion VOIs were placed by trained non-clinicians under supervision; although ICCs were high, subtle selection bias cannot be excluded. Third, qualitative review relied on one experienced reader; multi-reader validation would bolster generalisability. Fourth, SUVs are semi-quantitative and context-sensitive (uptake time, glucose status, body composition, scanner calibration). The paired design mitigates, but does not eliminate, these influences; external validity across vendors and software versions remains to be shown [19,20,28]. Fifth, this study was performed on a GE Discovery 710 PET/CT scanner, and results may vary across different scanner generations. Sixth, a phantom-based comparison was not performed. Seventh, all statistical testing was performed in Microsoft Excel, which carries some potential for error. Finally, we did not test adaptive or spatially varying penalisation schemes that might, in principle, extract further small-lesion gains, these remain investigational.

Future work

Three strands appear most impactful. First, prospective, multi-centre validation should be undertaken to harmonise acquisition parameters (uptake time windows, bed durations) and reconstruction protocols (fixed β), include multi-reader assessments with forced-choice preference testing, and integrate phantom cross-calibration so that β ≈ 800 on one platform truly maps to β ≈ 800 on another [19,25]. Second, outcome-linked analyses are needed to test whether BSREM-derived metrics (e.g., lesion SUVₘₐₓ at β ≈ 800) correlate more strongly with clinically proximate endpoints, such as PRRT eligibility, PRRT response categorisation, or progression-free survival, than OSEM-derived comparators [21,24]. Finally, method development should explore lesion-size-aware or adaptive penalisation and assess interactions with time-of-flight (TOF) kernels or point-spread function (PSF) models, while quantifying how scanner or software updates shift the SNR/SUV operating point so that services can re-tune β with minimal friction.

Summary

Penalised-likelihood reconstruction (BSREM; Q.Clear) enhances lesion SUVₘₐₓ and SNR for ^68^Ga-DOTATATE PET/CT in NETs compared with OSEM (VPFX), while preserving lesion-to-liver SBR and stabilising parenchymal backgrounds. The β ≈ 800 setting emerges as a practical default that aligns objective gains with subjective preference. With disciplined VOI practice and routine QA, these benefits appear observer-robust and immediately translatable to clinical reporting. Larger, multi-centre, and outcome-linked studies should now confirm external validity and define the extent to which reconstruction-level optimisation improves decision-making and patient outcomes.

## Conclusions

In this retrospective paired study of ^68^Ga-DOTATATE PET/CT for NETs, BPL (Q.Clear) improved lesion SUVₘₐₓ and SNR compared with OSEM (VPFX), without materially altering lesion-to-liver contrast. Qualitative assessment favoured a penalisation factor around β ≈ 800, and inter-observer agreement was excellent, supporting the robustness of measurement.

These findings support adopting Q.Clear with a penalisation factor near β ≈ 800 for NET imaging to enhance lesion conspicuity while preserving stable semi-quantification. Given the single-centre design, modest sample size, and single-reader qualitative assessment, prospective multicentre validation with outcome correlation is warranted. Standardising acquisition and reconstruction, explicitly including the penalisation factor, should facilitate harmonised reporting and reliable longitudinal follow-up.
